# Dysglycemias in pregnancy: from diagnosis to treatment. Brazilian consensus statement

**DOI:** 10.1186/1758-5996-2-27

**Published:** 2010-04-24

**Authors:** Carlos Antonio Negrato, Renan M Montenegro, Rosiane Mattar, Lenita Zajdenverg, Rossana PV Francisco, Belmiro Gonçalves Pereira, Mauro Sancovski, Maria Regina Torloni, Sergio A Dib, Celeste E Viggiano, Airton Golbert, Elaine CD Moisés, Maria Isabel Favaro, Iracema MP Calderon, Sonia Fusaro, Valeria DD Piliakas, José Petronio L Dias, Marilia B Gomes, Lois Jovanovic

**Affiliations:** 1 Gestational Diabetes Department of the Brazilian Diabetes Society, São Paulo-SP, Brazil; 2 School of Medicine of the Federal University of Ceará, Fortaleza-Ce, Brazil; 3 Federal School of Medicine of São Paulo State (UNIFESP), São Paulo-SP, Brazil; 4 Federal University of Rio de Janeiro, Rio de Janeiro-RJ, Brazil; 5 School of Medicine of São Paulo University (USP), São Paulo-SP, Brazil; 6 School of Medical Sciences of Campinas (UNICAMP), Campinas-SP, Brazil; 7 School of Medicine of ABC, ABC-SP, Brazil; 8 Nutrition Department of the Brazilian Diabetes Society, São Paulo-SP, Brazil; 9 Federal University of Health Sciences of Porto Alegre (UFRGS), Porto Alegre-RS, Brazil; 10 School of Medicine of Ribeirão Preto (USP), Ribeirão Preto-SP, Brazil; 11 Brazilian Diabetes Society, Jundiaí-SP, Brazil; 12 Botucatu Medical School, São Paulo State University (UNESP), Botucatu-SP, Brazil; 13 Hospital and Maternity Leonor Mendes de Barros, and UNICASTELO, São Paulo-SP, Brazil; 14 Santa Isabel Maternity, Bauru-SP, Brazil; 15 Endocrine and Diabetes Unit, State University of Rio de Janeiro, Rio de Janeiro-RJ, Brazil; 16 Sansum Diabetes Research Institute, Santa Barbara, California, USA

## Abstract

There is an urgent need to find consensus on screening, diagnosing and treating all degrees of DYSGLYCEMIA that may occur during pregnancies in Brazil, considering that many cases of DYSGLYCEMIA in pregnant women are currently not diagnosed, leading to maternal and fetal complications. For this reason the Brazilian Diabetes Society (SBD) and the Brazilian Federation of Gynecology and Obstetrics Societies (FEBRASGO), got together to introduce this proposal. We present here a joint consensus regarding the standardization of clinical management for pregnant women with any degree of Dysglycemia, on the basis of current information, to improve medical assistance and to avoid related complications of Dysglycemia in pregnancy to the mother and the fetus. This consensus aims to standardize the diagnosis among general practitioners, endocrinologists and obstetricians allowing the dissemination of information in basic health units, public and private services, that are responsible for screening, diagnosing and treating disglycemic pregnant patients.

## 1. Introduction

The organization of the health professions into specialties and subspecialties according to body organs and systems is often more pragmatic than scientific. The human organism is a single unit composed of a seemingly infinite number of biologic processes so intertwined that abnormalities of almost any of its parts or processes have profound effects on multiple other body areas, exemplified in this document by the common and complex theme of Dysglycemia in pregnancy.

The aim of this document is to provide health professionals, especially endocrinologists, obstetricians and general practitioners a better understanding of the current consensus on screening, diagnosing and treating all degrees of Dysglycemia that may occur during pregnancies in Brazil, considering that many cases are currently not diagnosed and consequently not treated leading to maternal and fetal poor outcomes.

This is a joint consensus of the Brazilian Diabetes Society (SBD) and the Brazilian Federation of Gynecology and Obstetrics Societies (FEBRASGO) regarding the standardization of clinical management for pregnant women with any degree of Dysglycemia, on the basis of current information, to improve medical assistance and to avoid related complications of Dysglycemia in pregnancy to the mother and the fetus.

## 2. Dysglycemias in pregnancy

Dysglycemia is currently the most prevalent metabolic alteration found during pregnancy [1]. Its prevalence during pregnancy can be found in up to 13% of pregnant women. The occurrence of type 1 diabetes (T1D) in the pregnant population is of 0.1 %/year, of type 2 diabetes (T2D) is of 2 to 3 %/year and that of gestational diabetes (GD) is about 12-13 %, depending on the diagnostic criteria used and the studied population [2]. In Brazil, the prevalence of GD found by the Brazilian Study of Gestational Diabetes Working Group was of 7.6% [3-5].

It is very important to know the type of diabetes underlying the dysglycemic state, since it can have different impact in the course of pregnancy and in fetal development. Pre-gestational diabetes, both T1D or T2D, is more severe, because its effects start soon, during fertilization and ovule's implantation, affecting mostly the organogenesis, leading to a higher risk of precocious miscarriages, severe congenital malformations and fetal growth restriction, mainly in those cases that are not adequately controlled [6]. Besides fetal complications, maternal poor outcomes can also be relevant, especially in the presence of previous complications such as retinopathy and nephropathy [7].

GD is generally diagnosed in the second half of pregnancy and affects especially fetal growth [8]. Fetuses of mothers with GD have a greater risk to present with macrosomia and neonatal hypoglycemia. Also, obesity and impairment in the psychomotor development can occur later on during lifetime [9]. If diabetes is diagnosed before this period of pregnancy, it is probably a pre-existing diabetes of any type, that was present before pregnancy and was not diagnosed.

## 3. Recommendations for patients with pre-existing diabetes

### 3. 1. Preconception information for women with pre-existing diabetes

From adolescence onwards, advise women about the importance of avoiding unplanned pregnancy [6] (B). Inform women and their families about how diabetes affects pregnancy and how pregnancy affects diabetes [10]. Information and advice should cover the following points:

• The impact of poor glycemic control in the course of pregnancy and in the risks for the mother and the fetus.

• The role of diet, weight, and exercise (including weight loss advice for women who have a body mass index above 25 kg/m^2^).

• The increased risk of having a baby who is large for gestational age (LGA), increasing the likelihood of birth trauma, induction of labor, and cesarean section.

• The importance of maternal glycemic control during labor and birth and early feeding of the baby to reduce the risk of neonatal hypoglycemia.

• The possibility of transient morbidity (such as hypoglycemia or respiratory distress syndrome) in the baby during the neonatal period, which may require admission to a neonatal unit.

• The need for folic acid supplementation (600 mcg to 5 mg a day) until 12^th ^gestation week to reduce the risk of having a baby with a neural tube defect.

• The risks of hypoglycemia and of hypoglycemia unawareness during pregnancy and the effects of nausea and vomiting during pregnancy on glycemic control.

The need for assessment of diabetic retinopathy and nephropathy before, during and after pregnancy, and the risk of its worseningOffer preconception care and advice to women who are planning to become pregnant before they discontinue contraception. Inform them that establishing a good glycemic control before conception and continuing it throughout pregnancy reduces- but does not eliminate - the risks of miscarriage, congenital malformations, stillbirth, and neonatal death [6] (B).

Offer a structured continuous education program that will provide a better understanding of diabetes in pregnancy in what concerns diet, carbohydrate counting, correct insulin dose adjustment and self monitoring of blood glucose (SMBG), as soon as possible, to women that are planning to become pregnant. Assess the presence of nephropathy, neuropathy, retinopathy, cardiovascular disease, hypertension, dyslipidemia, depression and thyroid dysfunctions in women with diabetes who are contemplating pregnancy, and start its treatment as soon as possible [11].

### 3.2. Glycemic control before and during pregnancy

Advise women with pre-existing diabetes who are planning to become pregnant to keep their HbA_1c _concentration as close as possible to the normal range, if this is safely achievable, avoiding the occurrence of hypoglycemia [12]. The recommended level of HbA_1c _is below 6.0%, or up to 1% above the maximum value informed by the clinical analyses laboratory where the tests are performed. Preferably, HbA_1c _test should be performed using a Diabetes Control and Complications Trial (DCCT) - aligned assay [12] (B). Reassure women that any reduction in HbA_1c _towards the target of 6.0% is likely to reduce the risk of congenital malformations that occur more frequently when diabetes is not under control at the beginning of pregnancy. Women whose HbA_1c _is above 10% should be strongly advised to avoid pregnancy until they reach a better metabolic control, due to the high risk of fetal malformations and miscarriages [13]. Pregnancy should be planned when diabetes is under control and preferably with HbA_1c _values within the normal range.

HbA_1c _test should be performed, at the first antenatal visit, and then monthly until target levels <6% are achieved, and every 2-3 months thereafter. Self monitoring of blood glucose (SMBG) is a key component of diabetes therapy during pregnancy and should be included in the management plan. Daily SMBG, both before and after meals, at bedtime and occasionally between 2:00 A.M. to 4:00 A.M., will provide optimal results in pregnancy [14] (C). Fingerstick SMBG is better than alternate site testing in pregnancy, since it may not identify rapid changes in glucose concentrations characteristic of pregnant women with diabetes [14] (C).

Glycemic control during pregnancy is considered excellent when glycemic levels before meals, at bedtime and between 2:00 A.M. to 4:00 A.M are between 60 and 105 mg/dl (3.5 and 5.9 mmol/l), with a postprandial peak (measured one hour after beginning the meal) between 100 and 140 mg/dl (5.5 and 7.8 mmol/l). Women who present increased risk of hypoglycemia should have these targets during fasting state higher than 100 mg/dl (5.5 mmol/l). Glucose level 1-h after beginning the meal is the closest value to the highest postprandial peak measured by continuous glucose monitoring [14] (C).

### 3.3. Medical Nutrition therapy

Pregnant women with diabetes should receive individualized medical nutrition therapy to achieve treatment goals. Daily energy intake should be based on BMI, physical activity level, fetal growth pattern and the need to prevent excess maternal weight gain and post-partum weight retention [15]. The total recommended caloric content should be composed by:

- 40-45 % carbohydrates

- 15-20 % proteins (minimum 1.1 g/kg/day)

- 30-40 % fat

Diet must also be planned aiming to promote carbohydrate intake along the day, to achieve optimal glycemic control and to avoid hypoglycemia, hyperglycemia and ketonemia. In general it is necessary to divide the total daily food intake in 6 light meals [16] (C). Women that are on insulin treatment should receive special attention regarding insulin doses adequacy, the exact time of its administration and to the nutritional content of each meal. Bedtime meal should not be omitted and it has to have 25 g of complex carbohydrates, besides proteins or lipids to avoid hypoglycemia during the night.

The adjustment of prandial insulin can be done by carbohydrate counting every time the patient eats.

Nonnutritive sweeteners such as aspartame, saccharin, acesulfame- K and sucralose can be moderately used [16] (C).

### 3.4. Vitamins and trace elements supplementation

The supplementation of folic acid (600 μg to 5 mg a day) from preconceptional period until the closure of neural tube (12 weeks' gestation) is recommended for all pregnant women, including those who are diabetic, to reduce the risk of having a baby with a neural tube defect [17] (A).

Supplementation with other vitamins and trace elements should be done when nutritional deficiencies of these vitamins and trace elements are detected [15] (C).

### 3.5. Physical activity/exercise

The benefits of exercise for pregnant women include a sense of wellbeing, decreased weight gain, reduction of fetal adiposity, improved glucose control, and better tolerance of labor [18]. Physical activity reduces insulin resistance, which improves blood glucose utilization and consequently blood glucose control. This effect can avoid or postpone the need of insulin use in women with GD.

Physical activity of low intensity should be encouraged for women previously sedentary if they have no contraindications to practice it. Those already practicing exercise before pregnancy should maintain their physical activity if no contraindication is present. Contraindications for the practice of physical activity during pregnancy are:

✓ Pregnancy induced hypertension

✓ Premature membranes' rupture

✓ Symptoms of preterm labor

✓ Persistent uterine bleeding after the second trimester

✓ Intrauterine fetal growth restriction

✓ Nephrotic syndrome

✓ Preproliferative and proliferative retinopathy

✓ Hypoglycemia unawareness

✓ Advanced peripheral and dysautonomic neuropathy

When indicated, physical activities should be performed on most days of the week for a period of at least 30 minutes. Women should choose an activity that does not have a risk of falling down or cause an abdominal trauma, such as walking. The minimal target of 30 minutes can be divided into three 10-minutes sessions preferably after meals. Exercises must be conducted in an appropriate environment, without excessive heat, to avoid the risk of dehydration. Capillary blood glucose should be monitored before and after exercises. Physical activities should not increase blood pressure, cause uterine contractions and fetal growth restriction [19] (B).

### 3.6. Pharmacological management (insulins and safety of medications for diabetes and its complications before and during pregnancy)

Because insulin preparations tested to date have been determined not to cross or minimally cross the placenta, insulin has been the treatment of choice in most parts of the world for patients with gestational Dysglycemia. Patients are advised to discontinue all oral hypoglycemic agents, preferably before pregnancy or as soon as pregnancy is diagnosed and substitute with insulin, because of its tested safety and efficacy in glycemic control. Recent trials have shown the safety of metformin during pregnancy [20-22] (B) and of the use of glibenclamide in patients with GDM after the second trimester [23] (B). Glibenclamide crosses the placenta minimally; metformin crosses the placenta in significant amounts and although no adverse effects have been described, it seems prudent to obtain further data before glibenclamide and/or metformin become commonly prescribed during pregnancy.

For optimal glycemic control during pregnancy in women with pre-existing diabetes, intensified insulin regimens with multiple doses of subcutaneous long and short-acting insulins or continuous insulin infusion, usually give the best results. In women who were using insulin before pregnancy, it is generally necessary to reduce the dose in about 10 to 20% during the first trimester. Between the 18th and the 24th gestation week this dose must be increased. During the third trimester, the increased production of hormones by the placenta with antagonistic effects to insulin makes it necessary to increase insulin dose again, generally requiring doses that are the double or triple of those used before pregnancy. For converting women with T2D to insulin therapy, an initial total daily dose of 0.7-1.0 unit/kg actual body weight is often effective, adjusted according to subsequent blood glucose concentrations. After delivery, insulin requirements fall dramatically, and many times in the following days, insulin doses must be adjusted to half of those used at the end of pregnancy or return to pre-pregnancy doses. The rapid acting insulin analogs, such as insulin aspart and lispro are safe during pregnancy, leading to better postprandial glycemic levels and causing less hypoglycemia [24] (B). NPH human insulin is still the first choice for basal insulin [24] (A). There are not consistent data regarding the use of the long acting insulin analogs, detemir and glargine in pregnancy, although many isolated reports and data of some studies have shown promising results with their use (C). Continuous subcutaneous insulin infusion can be used when available. The ideal sites for insulin injections during pregnancy are the abdomen and hips [24] (C).

In conclusion, management of GD with medication, both oral and insulin, has been changing in the last years, but quite slowly. Metformin is a logical option for women with GD. It improves insulin sensitivity, probably by activating AMP kinase, and is not associated with weight gain or hypoglycemia, but randomized trials to assess the efficacy and safety of its use for GD are lacking. Recently a randomized trial was conducted in Australia and New Zealand, aiming exactly to assess its efficacy and safety in this condition. A group of 751 women with GD was randomly assigned at 20 to 33 weeks of gestation to open treatment with metformin (with supplemental insulin if required) or insulin. The primary outcome was a composite of neonatal hypoglycemia, respiratory distress, need for phototherapy, birth trauma, 5-minute Apgar score less than 7, or prematurity. The trial was designed to rule out a 33 % increase (from 30 to 40%) in this composite outcome in infants of women treated with metformin as compared with those treated with insulin. Secondary outcomes included neonatal anthropometric measurements, maternal glycemic control, maternal hypertensive complications, postpartum glucose tolerance, and acceptability of treatment. Of the 363 women assigned to metformin, 92.6% continued to receive metformin until delivery and 46.3 % received supplemental insulin. The rate of the primary composite outcome was similar in both groups; 32.0 % in the group assigned to metformin and 32.2 % in the insulin group. More women in the metformin group than in the insulin group stated that they would choose to receive their assigned treatment again (76.6 vs. 27.2 %). The rates of other secondary outcomes did not differ significantly between the groups. There were no serious adverse events associated with the use of metformin. So, it was concluded that in women with GD, metformin (alone or with supplemental insulin) is not associated with increased perinatal complications as compared with insulin. The women preferred metformin to insulin treatment [20].

The use of sulfonylureas in GD is also controversial, and rarely done, because of concern about teratogenicity and hypoglycemia. There is also little information about the efficacy of these drugs in this group of women. So, a large clinical trial studied 404 women with singleton pregnancies and GD that required treatment. The women were randomly assigned between 11 and 33 weeks of gestation to receive glibenclamide or insulin according to an intensified treatment protocol. The primary end point was achievement of the desired level of glycemic control. Secondary end points included maternal and neonatal complications. The mean (+/-SD) pretreatment blood glucose concentration as measured at home for one week was not statistically different: 114+/-19 mg/dl (6.4+/-1.1 mmol/l) in the glibenclamide group and 116+/-22 mg/dl (6.5+/-1.2 mmol/l) in the insulin group. The mean concentrations during treatment were also not statistically different: 105+/-16 mg/dl (5.9+/-0.9 mmol/l) in the glibenclamide group and 105+/-18 mg/dl (5.9+/-1.0 mmol/l) in the insulin group. Eight women in the glibenclamide group (4 %) required insulin therapy. There were no significant differences between the glibenclamide and insulin groups in the percentage of infants who were large for gestational age (12 and 13 %, respectively); who had macrosomia, defined as a birth weight of 4000 g or more (7 and 4 %); who had lung complications (8 and 6 %); who had hypoglycemia (9 and 6 %); who were admitted to a neonatal intensive care unit (6 and 7 %); or who had fetal anomalies (2 and 2 %). The cord-serum insulin concentrations were similar in the two groups, and glibenclamide was not detected in the cord serum of any infant in the glibenclamide group. It was concluded then that in women with GD, glibenclamide is a clinically effective alternative to insulin therapy [23].

The decision to begin insulin therapy may be a choice for a gestational diabetic woman and her physician when the medical nutrition therapy is too difficult to maintain glycemic levels in acceptable ranges. Those with pregestational diabetes generally have to change their insulin regimen. Depending on the type, severity, and stage of diabetes, patients may have only elevated postprandial glucose levels and normal fasting blood glucose levels, or the fasting glucose levels may be elevated as well. If postprandial glucose is the target of treatment, the rapid-acting insulin analogs lispro and aspart, appear to be as safe and effective as regular human insulin, achieving better postprandial glucose concentrations with less late prandial hypoglycemia. If the patient has elevated fasting and postprandial blood glucose levels and requires multiple daily injections to achieve good glycemic control, a basal-bolus regimen should be considered. The long-acting insulin analogs do not have as pronounced a peak effect as NPH insulin and therefore cause less nocturnal hypoglycemia. However, the safety of these insulin analogs needs to be further established in pregnant women. Issues that will need to be further clarified include the question of whether these insulin analogs have teratogenic effects on the developing fetus, alter the balance between the binding affinity to IGF-I receptor and insulin receptor, are associated with increased risk of retinopathy, or show increased antibodies levels. Because the lack of information, large-scale controlled clinical trials are warranted [24].

Discontinue angiotensin converting enzyme inhibitors and angiotensin II receptor antagonists, due to its association with embriopathies and fetopathies, before pregnancy or as soon as pregnancy is confirmed, and substitute them with alternative anti-hypertensive agents suitable for use during pregnancy [25,26] (A). These agents are: methyldopa, long acting nondihydropyridine calcium channel blockers and beta blockers with partial beta agonist activity like carvedilol, labetalol and pindolol; the use of atenolol has been associated with fetal growth restriction and must be avoided [27] (C).

Discontinue statins before pregnancy or as soon as pregnancy is confirmed, due to its potential teratogenic effects [28,29] (A).

It is still not clear if the use of fibrates in pregnancy is safe or not; it should be used only in cases of severe hypertrygliceridemia, when no response to diet is met and if there is an increased risk of developing acute pancreatitis [27] (D).

### 3.7. Management of diabetic emergencies and complications during pregnancy

Advise and teach women with insulin treated diabetes about the risks of hypoglycemia and how to prevent it, particularly during the night and during the sleeping hours. Advise women, their partners and other family members about these risks and empower them to give the first aid without delay [24] (B).

Exclude diabetic ketoacidosis as a matter of urgency in women with T1D who present with any infectious disease, dehydration and high glycemic levels [10] (D).

Offer retinal and renal assessment to women with pre-existing diabetes before, during and after pregnancy because diabetic retinopathy may worsen during pregnancy and diabetic nephropathy is associated with adverse pregnancy outcomes such as intrauterine growth restriction, preeclampsia and preterm birth. The risk of worsening a proliferative retinopathy is extremely high in those women that did not receive previous laser therapy. Ischemic coronary disease, if not treated is associated to high mortality rates [10] (B).

## 4. Recommendations for patients with gestational diabetes

Gestational diabetes (GD) is defined as carbohydrate intolerance of varying degrees of severity with onset or first recognition during pregnancy, regardless of whether insulin is used for treatment or the condition persists after pregnancy. It does not exclude the possibility that unrecognized glucose intolerance may have antedated the pregnancy [30]. The importance of diagnosing diabetes during pregnancy was reinforced by the fact that a greater frequency of miscarriages, macrosomia and perinatal mortality was found in the offspring of women that developed diabetes during pregnancy, when compared to the control group [30]. It generally represents the early onset of T2D that occurs during pregnancy and has many risk factors such as:

- Advanced ages (generally = 35 years)

- Overweight, obesity (BMI = 25) before pregnancy or in the first trimester and/or excessive weight gain in the index pregnancy [31,32]

- Family history of diabetes in first degree relatives

- Excessive fetal growth (macrosomia or large for gestational age [LGA] fetuses), polyhydramnio, hypertension or preeclampsia in the index pregnancy

- Poor obstetric history: previous miscarriages, congenital malformations, fetal or neonatal death, macrosomia or gestational diabetes

- Polycystic ovaries syndrome

- Use of drugs that can cause hyperglycemia, like thiazidic diuretics, corticosteroids, excessive doses of thyroid hormones etc...

**Figure 1:**
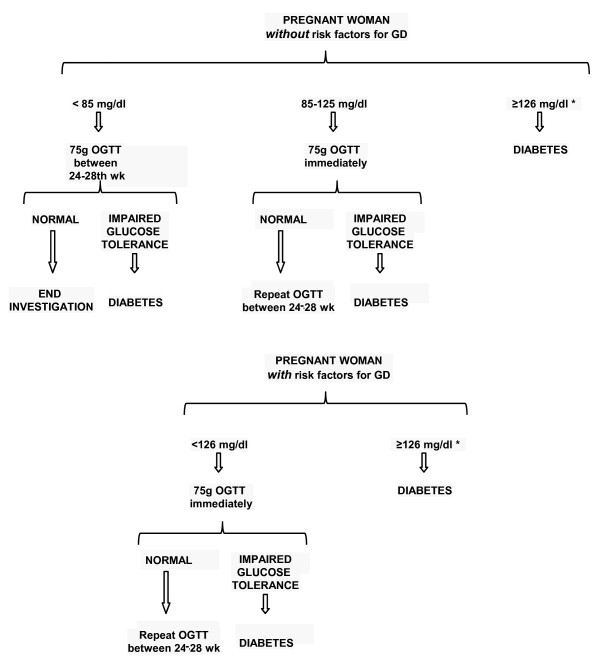
**Algorithm for the diagnosis of gestational diabetes**. * Confirmed with a 2nd abnormal value.

There is great controversy about the indication of screening for GD in the literature. The majority of recommendations come from consensus done by groups of specialists [10] (D). In the present moment, current recommendations are evidence-based; the screening for GD must be universal, what means that all pregnant women must be investigated. All women should have their plasma glucose level measured at the first antenatal visit. If fasting glycemia is = 85 mg/dl (4.7 mmol/l), an oral glucose tolerance test should be performed immediately in order to detect a pre-existing, non diagnosed diabetes. If the test is normal, it should be repeated between the 24^th^-28^th ^gestation weeks (Figure 1).

For the diagnosis of GD, the three hour 75 g oral glucose tolerance test (OGTT) must be performed between the 24^th ^- 28th gestation weeks. The American Diabetes Association's diagnostic criteria for GD, use the cutoff points suggested by Carpenter & Coustan, with blood glucose levels = 95 mg/dl (5.2 mmol/l, = 180 mg/dl (10.0 mmol/l) and = 155 mg/dl (8.6 mmol/l), at fasting, 1 and 2 hrs, respectively; two or more altered points lead to the diagnosis of GD (Table 1) [4,33]. Recently, in a workshop for experts that took place in Pasadena, California in June 2008, sponsored by the International Association of Diabetes and Pregnancy Study Groups (IADPSG), it was decided that the GD diagnosis criteria should be based on the findings of the HAPO study, an observational trial that aimed to clarify the accurate glucose threshold that links maternal hyperglycemia and adverse perinatal outcomes [34]. It was then suggested new cutoff points for fasting, 1 and 2 hrs of blood glucose = 92 mg/dl (5.1 mmol/l), ≥180 mg/dl (10.0 mmol/l) and ≥153 mg/dl (8.5 mmol/l), respectively. It was then, discussed the possibility that only one value above the normal could lead to the diagnosis of GD (ADA 2009, not published). These criteria were recently approved by the International Diabetes Federation (IDF 2009, not published) (Figure 1).

**Table 1:** Diagnosis of gestational diabetes using 75 g Oral Glucose Tolerance Test.

	ADA, SBD, and FEBRASGO*	International Association of Diabetes and Pregnancy Study Groups (IADPSG) (ADA and IDF meetings 2009 -unpublished data)**
Fasting	≥95 mg/dl (5.27 mmol/l)	≥92 mg/dl (5.11 mmol/l)
1 hr	≥180 mg/dl (10.0 mmol/l)	≥180 mg/dl (10.0 mmol/l)
2 hrs	≥155 mg/dl (8.61) mmol/l)	≥153 mg/dl (8.5) mmol/l)

* Two altered values are considered diagnostic of GDM

** One altered value is considered diagnostic of GDM

It is of importance to notice that these tests must be performed three days after the consumption of a diet containing more than 150 g of carbohydrates daily; the patient cannot smoke during the test and must stay seated or laid down.

Fasting glycemia of 100 mg/dl (5.5 mmol/l), considered the upper level for normal adults is not valid in pregnancy. Diagnosis of GD should not be done with random blood glucose (except when the levels of glycemia are ≥200 mg/dl [11.1 mmol/l]), with 50 g glucose challenge test or urine analyses for glucose [10] (B).

Inform women with GD about the effects of GD on pregnancy; tell them that good glycemic control throughout pregnancy will reduce the risks of fetal macrosomia, trauma during birth (to themselves and the baby), induction of labor or cesarean section, neonatal hypoglycemia, and perinatal death. Instruct them on self monitoring blood glucose. Targets for blood glucose control are the same as for women with pre-existing diabetes.

Recent studies have evidenced that intervention in women with GD can prevent the occurrence of many adverse outcomes [35] (B), even in those women presenting with less severe degrees of Dysglycemia, that are not considered as diagnostic of GD [31,32] (A). The initial approach to GD consists in tailoring a diet that will allow adequate weight gain and normalization of glycemia. The total caloric amount of the diet should lead to a weight gain of about 300-400 g per week after the second trimester of pregnancy [15].

Regular physical activities are part of the treatment of GD, taking always in account the presence of any kind of obstetric contraindication [18] (B).

Pharmacological therapy must be considered if diet and exercise fail to maintain blood glucose targets during a period of one to two weeks (fasting = 95 mg/dl [5.2 mmol/l] and 1 hr postprandial ≥140 mg/dl [7.8 mmol/l]) [36] (B). Glycemic control should be done with one fasting and two postprandial glycemias performed daily or at least weekly, when there exists no conditions for self monitoring of blood glucose. Excessive fetal growth, observed in an ultrasound investigation done between the 29th and 33rd gestation weeks, that shows a fetal abdominal circumference above the 70th centile can be used to consider pharmacological therapy [37] (B). The standard hypoglycemic therapy approach is the addition of exogenous insulin. Starting doses average 0.6-1.0 units/kg depending on the stage of gestation. Combinations of intermediate or long acting preparations with short or rapid-acting preparations are useful to reach glucose targets and improve fetal outcomes. Insulin frequently is stopped after delivery. It is necessary reassess maternal glucose levels post partum to evaluate if the patient persisted dysglycemic, and if so, it is necessary to identify the type of diabetes that is present.

## 5. Antenatal care appointments

Offer pregnant women with diabetes, either pre-existing or GD an education program provided by a diabetes care team.

Antenatal appointments should cover care specifically for women with diabetes in addition to the care that is provided routinely for healthy pregnant women.

Assessment of glycemic control should be performed every one to two weeks by the physician and/or other members of the diabetes care team.

Prioritize women with pregestational diabetes to perform a fetal echocardiography for evaluation of the four chamber of fetal heart, aiming to visualize any kind of anatomic or functional dysfunction [25] (A).

The aims of fetal evaluation are to observe vitality in the first trimester, structural integrity in the second trimester and monitor fetal growth and wellbeing in the third trimester (Table 2).

**Table 2:** Recommendations for fetal evaluation in pregnancy complicated by diabetes.

1st Trim.	US to evaluate gestational age/nuchal transparency thickness and screen fetal malformations
**2nd Trim.**	Morphologic US to screen malformations - 20-24th gestation week Uterine and umbilical arteries doppler - 20th gestation week Fetal Echocardiogram - 24-26^th ^gestation week (in preexisting diabetes) US monthly 24th gestation week onwards to evaluate fetal growth and polyhydramnio

**3rd Trim.**	Monthly US until delivery. In case of fetal growth restriction or LGA, it should be performed every two weeks Basal CTG between 24-28^th ^gestational week in cases of preexisting diabetes Umbilical arteries doppler in the presence of hypertension, preeclampsia or vasculopathy. Monthly US from 24^th ^gestational week to evaluate fetal growth and the presence of polyhydramnios Daily Fetal movements count - 3×/day after 28^th ^gestation weeks with patient on left side decubitus

* US = Ultrasound ** LGA = Large for gestational age *** CTG = Cardiotocography

Those women who present with poor glycemic control and/or with hypertension, should have anticipated and performed in shorter periods of time those tests that evaluate fetal wellbeing, since the risk of fetal death is proportional to the degree of maternal hyperglycemia and hypertension.

## 6. Preterm labor in women with diabetes

Antenatal steroids for fetal lung maturation are not contraindicated but should be administered with additional insulin in women with insulin treated diabetes [10] (D).

Tocolysis for inhibition of preterm labor is not contraindicated, but do not use betamimetics for tocolysis in women with diabetes [10] (D).

## 7. Timing and mode of birth

Diabetes is not an absolute indication for cesarean section. In women with diabetes with a good metabolic control the mode of birth should be vaginal.

An anesthetic assessment can be offered to relieve labor's pain, especially in women with co-morbidities such as obesity and autonomic neuropathy.

Capillary blood glucose levels should be monitored every hour during labor and birth and in the post anesthetic period.

Offer elective, through induction of labor or, if indicated, elective cesarean section, when there is maternal or fetal indication [10] (D).

## 8. Glycemic control during labor and birth

Capillary blood glucose should be monitored hourly during labor and birth to maintain glucose levels between 70 and 140 mg/dl (3.88 and 7.8 mmol/l); if the concentration is not maintained in this range, use continuous intravenous dextrose and insulin infusion [10] (D).

In women with DM1, consider intravenous dextrose and insulin infusion from the onset of labor [10] (D). Women that use insulin pumps for continuous insulin infusion must change their basal/bolus ratios, depending on the type of labor.

## 9. Initial assessment and care of the newborn baby

Advise women to give birth in hospitals where advanced neonatal resuscitation skills are available 24 hours a day; keep babies with their mothers unless a clinical complication or abnormal clinical signs arise that warrant admission for an intensive or special care unit [10] (A).

Feed babies as soon as possible after birth (within 30 minutes) and then every two to three hours until feeding maintains prefeed blood glucose concentrations of at least 40 mg/dl (2.22 mmol/l). Test blood glucose concentrations at two to four hours after birth. Only if concentrations are below 40 mg/dl (2.22 mmol/l) in two consecutive readings despite maximal support for feeding, or if there are abnormal clinical signs, or if the baby will not feed orally effectively, should additional measures such as tube feeding or intravenous dextrose be given. Test blood glucose in babies who present with clinical signs of hypoglycemia (such as abnormal muscle tone or low level of consciousness, fits, apnea) and start treatment with intravenous dextrose as soon as possible [10] (A).

Perform echocardiography for babies who show clinical signs associated with congenital heart disease (including heart murmur) or cardiomyopathy.

Do not test for polycythemia, hyperbilirubinemia, hypocalcaemia, or hypomagnesaemia unless the baby has clinical signs.

Recognize criteria for admission to a neonatal unit, such as hypoglycemia associated with abnormal clinical signs, respiratory distress, signs of cardiac decompensation, or neonatal encephalopathy.

## 10. Postnatal management of diabetes

### 8.1. Pre-existing diabetes

• Breastfeeding should be encouraged. Exclusive breastfeeding is the ideal nutrition, and provides protection against infections in infants [38] (A).

• Reduce insulin immediately after the birth in women with insulin treated pre-existing diabetes. Monitor blood glucose levels carefully to establish the appropriate dose, and inform women of the increased risk of hypoglycemia in the postnatal period, especially if breastfeeding (It is advisable to eat a meal or snack before or during feeds) [15] (D).

Resume (or continue) use of metformin and glibenclamide immediately after birth in women with pre-existing T2D diabetes who are breastfeeding. Only 0.4% of metformin dose taken by the mother is detected in maternal milk, and this is not dependent on the time the drug was taken.

• Studies done with small number of cases (maximum 9 children), did not detect the drug in the babies [39-41]. Glibenclamide and glipizide were not detected in maternal milk and no hypoglycemia was reported in the babies, although the number of studied cases was small [42]. (Table 3).

**Table 3:** Use of anti-diabetic medications in women with diabetes during lactation.

Drug	Milk transfer	Permission to use during lactation	Reference
Glibenclamide	No	Yes	[42]
Glicazide	Unknown	No	Not published
Glipizide	No	Yes	[42]
Glimepiride	Unknown	No	Not published
Metformin	Less than1%	Yes	[39-41]
Acarbose	Less than 2%	No	http://www.fda.gov
Rosi and pioglitazone	Rosi detected in milk from lactating rats. Pio not published	No	http://www.fda.gov
Sita and vildagliptine	Sitagliptin is secreted in the milk of lactating rats at a milk to plasma ratio of 4:1. It is not known whether sitagliptin is excreted in human milk. Vilda not published	No	http://www.fda.gov
Exenatide	Unknown	No	Not published

• Refer women with pre-existing diabetes back to their routine diabetes care arrangements; remind them of the importance of contraception and the need for preconception care when planning future pregnancies.

### 8.2. Gestational diabetes

• Discontinue insulin therapy immediately after the birth in women who were diagnosed with GD; test their blood glucose to exclude persisting hyperglycemia before transfer to community care, and remind them of the symptoms of hyperglycemia.

• Offer lifestyle advice (on weight control, diet, and exercise) and an OGTT performed with 75 g glucose load six weeks after delivery, adopting WHO's criteria for diabetes out of pregnancy, that is a fasting glycemia = 126 mg/dl (7.0 mmol/l) and/or glycemia = 200 mg/dl (11.1 mmol/l) two hours after the glucose load. Patients who present a fasting glycemia between 100 mg/dl (5.5 mmol/l) and 125 mg/dl (6.94 mmol/l) are classified as having an impaired fasting glucose (IFG), and those who present a glycemia between 140 mg/dl (7.8 mmol/l) and 199 mg/dl (11.06 mmol/l) two hours after glucose load, are considered as having an impaired glucose tolerance (IGT). If the test is normal consider performing at least a fasting plasma glucose measurement annually thereafter (B). Levels of HbA_1c _higher than 6.5% should be considered as diagnostic of diabetes [43]. Provide information about the risk of GD in future pregnancies, offer screening for diabetes when planning future pregnancies, and indicate early OGTT in future pregnancies and also self monitoring blood glucose.

### 8.3. Treatment of co-morbidities after delivery

• *Lipid Therapy: *Statins and fibrates should not be used during breastfeeding because they pass through breast milk and due to the potential for adverse effects to the infant (manufacturers' reports). When triglycerides levels are above 1000 mg/dl (55.55 mmol/l) in the presence of an appropriate diet, due to the high risk of pancreatitis, niacin, fish oil (free of mercury) or quitting breastfeeding should be considered.

• *Antihypertensive Therapy: *Use of ACE inhibitors, calcium channel blockers, low dose thiazides and Methyldopa during breastfeeding, even being transferred to the milk in small amounts, are safe [27]. Atenolol has been associated with bradycardia and hypotension in infants [44]. Propranolol and metoprolol could be indicated but infants should be monitored for manifestations of beta-blockade (C).

## 11. Contraception

Contraceptive counseling is an effective method for avoiding the undesirable consequences of an unplanned diabetic pregnancy. No one contraceptive method is appropriate for all women with diabetes, and counseling must be individualized.

If an oral contraceptive is the best choice, a low-dose combined (estrogen+ progestin) or sequential pill with ≤35 μg estrogen and a new progestin (levonorgestrel, desogestrel, gestodene, or norgestimate) low dose may be better, but the risk of cardiovascular effects must be considered. Progestin-only pills offer an alternative, but there is the possibility of elevated blood lipid levels and other effects.

Use of long acting injectable progestin is no longer recommended for diabetic patients.

Cooper-containing intrauterine devices appear to expose diabetic women to greater risk of infection when compared to non diabetic women.

Barrier methods such as diaphragm plus spermicide or condom plus foam, present high failure rate.

Rhythm increases failure rate, since diabetic women may not have regular menstrual cycles.

Once childbearing is completed, permanent sterilization of the diabetic woman or her mate may offer an acceptable means to prevent unplanned pregnancy compared with other contraceptive methods [45].

## 12. Final considerations

Drugs that can be used by pregnant diabetic women during pregnancy and breastfeeding can be seen in Table 4. The most important items highlighted in this consensus can be seen in Table 5, with the evidence levels of the main recommendations and conclusions.

**Table 4:** Safety for use of common prescribed drugs for women with diabetes during pregnancy and breastfeeding.

Drug	Safety for use during pregnancy	Safety for use during lactation	Level of evidence
**SWEETENERS**			
aspartame, saccharin, acesulfame-K and sucralose	Moderately	moderately	C
**ORAL ANTIHYPERGLICEMIC AGENTS**			
Glibenclamide	No consensus	Yes	B
Glicazide	No	No	B
Glipizide	No	Yes	B
Glimepiride	No	No	B
Metformin	No consensus	Yes	B
Acarbose	No	No	C
Rosi and pioglitazone	No	No	C
Sita and vildagliptin	No	No	C
Exenatide	No	No	D
**INSULIN**			
NPH	Yes	Yes	A
Regular	Yes	Yes	A
Lispro	Yes	Yes	B
Aspart	Yes	Yes	B
Gargine	No consensus	No consensus	C
Detemir	No consensus	No consensus	C
**ORAL ANTIHYPERLIPIDEMIC AGENTS**			
Gemfibrozil	No	No	A
Statins	No	No	A
**ANTIHYPERTENSIVE**			
Enalapril	No	With caution	A
Captopril	No	No	A
Lisinopril	No	No	A
Methyldopa	Yes	Yes	A
Losartan	No	With caution	A
Candesartan	No	No	A
Hydrochlorothiazide (low doses)	Yes	Yes	C
Calcium channel Inhibitors	No	Yes	C
Beta- blockers(labetalol, metoprolol, propanolol)	Yes	Yes	B
Atenolol	No	No	A
**THYROID HORMONES**			
Levothyroxine	Yes	Yes	A
**ANTITHYROID DRUGS**			
Methimazole	With caution	Yes	B
Propiltiouracil	Yes	Yes	B
Iodine	No	No	A
**ANTIDEPRESSANTS**			
Fluoxetine	No	No	B
Paroxetine	With caution	Yes	B
Tricyclic(amytriptyline, nortriptyline, clomipramine)	With caution	Yes	B
**ANTI-INFLAMMATORY DRUGS**			
Nimesulide	With caution	With caution	B
Mefenamic acid, ketoprofen, diclofenac, ibuprofen, meloxicam	With caution	Yes	B
**Analgesics**			
Acetaminophen	Yes	Yes	B
**Antibiotics**			
Quinolones(norfloxacin, moxifloxacin, ciprofloxacin)	No	No	C

**Table 5:** Evidence levels of the main recommendations and conclusions.

Recommendations and Conclusions	Evidence Level
• Diabetic patients must start pregnancy in ideal metabolic conditions (HbA_1c _< 6% or 1% above the maximum value used by the clinical analyses laboratory).	**B**

• Advise patients to self monitor capillary blood glucose before and after meals, at bedtime and sporadically between 2:00 and 4:00 AM.	**C**

• The amount of calories prescribed must be based on BMI. The total caloric amount recommended must be composed by: 40 to 45% carbohydrates, 15-20% proteins (minimum of 1, 1 mg/kg/day) and 30-40% fat.	**B**

• Use of folic acid before pregnancy until neural tube closure is recommended for all women including those with diabetes.	**A**

• Regular practice of physical activity will cause a wellbeing sensation, less weight gain, reduction in fetal adiposity, a better glycemic control and fewer problems during labor. Physical activity is contraindicated in case of: Pregnancy induced hypertension, premature membranes' rupture, preterm labor, persistent uterine bleeding after the second trimester, intrauterine growth restriction, nephrotic syndrome, pre and proliferative retinopathy, hypoglycemia unawareness, advanced peripheral neuropathy and dysautonomia	**A**

• In most parts of the world the recommendation is to discontinue the use of antidiabetic oral agents and its substitution for insulin, before pregnancy or soon after its diagnosis. Recent trials have shown the safety of metformin during pregnancy and the use of glibenclamide in patients with GD after the second trimester.	**B**

• Rapid acting insulin analogs such as insulin aspart and lispro are safe during gestation, lead to a better control of postprandial levels of glycemia and lower frequency of hypoglycemia. NPH human insulin is still the first choice among those intermediate acting insulins. There are some studies and short communications with the use of long acting insulin analogs detemir and glargine, but more consistent studies are warranted.	**A**

• Discontinue the use of angiotensin converting enzyme inhibitors, of angiotensin II receptor agonists and statins before pregnancy or as soon as it is confirmed, due to its association with embriopathies and fetopathies	**A**

• In order to simplify the diagnosis of GD, a fasting glycemia must be performed in the first antenatal visit. If glycemic level is ≥85 mg/dl and the patient shows risk factors for GD, a 75 g OGTT must be performed. If the test is normal, it must then be repeated between 24^th ^and 28^th ^gestation weeks.	**A**

• Diagnosis of GD should not be done with a random glycemia, with a 50 g glucose challenge test and urine glucose testing. Between 24-28^th ^gestation weeks, a fetal echocardiography should be performed to evaluate the four fetal heart chambers, aiming to diagnose any kind of anatomic or functional dysfunction.	**B**

• Perform an OGTT six weeks after delivery, and then, at least a fasting glycemia annually.	**B**

## Competing interests

The authors declare that they have no competing interests.

## Authors' contributions

All authors participated equally in the development of this Consensus;

All authors also read and approved the final manuscript.

## Authors' information section

Participants (Technical panel) section:

**Carlos Antonio Negrato - **Gestational Diabetes Department of the Brazilian Diabetes Society, São Paulo-SP, Brazil. carlosnegrato@uol.com.br** Disclosure Statement: **None

**Renan Magalhães Montenegro Junior - **School of Medicine of the Federal University of Ceará, Fortaleza-Ce, Brazil. renanjr@ufc.br** Disclosure Statement: **None

**Rosiane Mattar - **Federal School of Medicine of São Paulo State (UNIFESP), São Paulo-SP, Brazil. rosiane.mattar@globo.com** Disclosure Statement: **None

**Lenita Zajdenverg - **Federal University of Rio de Janeiro, Rio de Janeiro-RJ, Brazil. lenitaz@hucff.ufrj.br** Disclosure Statement: **None

**Rossana Pulcineli Vieira Francisco - **School of Medicine of São Paulo University (USP), São Paulo-SP, Brazil. rossana.francisco@hcnet.usp.br **Disclosure Statement: **None

**Belmiro Gonçalves Pereira - **School of Medical Sciences of Campinas (UNICAMP), Campinas-SP, Brazil. belmirop@hotmail.com** Disclosure Statement: **None

**Mauro Sancovski - **School of Medicine of ABC, ABC-SP, Brazil. maurosancovski@gmail.com** Disclosure Statement: **None

**Maria Regina Torloni - **Federal School of Medicine of São Paulo State (UNIFESP), São Paulo-SP, Brazil. rtorloni@uol.com.br **Disclosure Statement: **None

**Sergio Atalla Dib - **Federal School of Medicine of São Paulo State (UNIFESP), São Paulo-SP, Brazil. sergio.dib@unifesp.br** Disclosure Statement: **None

**Celeste Elvira Viggiano - **Nutrition Department of the Brazilian Diabetes Society, São Paulo-SP, Brazil. celeste.viggiano@imes.edu.com **Disclosure Statement: **None

**Airton Golbert - **Federal University of Health Sciences of Porto Alegre (UFRGS), Porto Alegre-RS, Brazil. agolbert@terra.com.br** Disclosure Statement: **None

**Elaine Christine Dantas Moisés - **School of Medicine of Ribeirão Preto (USP), Ribeirão Preto-SP, Brazil. elainemoises@hcrp.usp.br **Disclosure Statement: **None

**Maria Isabel Favaro - **Brazilian Diabetes Society, Jundiaí-SP, Brazil. belfavaro@hotmail.com** Disclosure Statement: **None

**Iracema de Mattos Paranhos Calderon - **Botucatu Medical School, São Paulo State University (UNESP), Botucatu-SP, Brazil. calderon@fmb.unesp.br** Disclosure Statement: **None

**Sonia Fusaro - **Federal School of Medicine of São Paulo State (UNIFESP), São Paulo-SP, Brazil. sfusaro@globo.com** Disclosure Statement: **None

**Valeria Diniz Duarte Piliakas - **Hospital and Maternity Leonor Mendes de Barros, and UNICASTELO, São Paulo-SP, Brazil. piliakcas@uol.com.br** Disclosure Statement: **None

**José Petronio Lourenço Dias - **Santa Isabel Maternity, Bauru-SP, Brazil. jpldias@hotmail.com** Disclosure Statement: **None

**Marilia Brito Gomes - **Endocrine and Diabetes Unit, State University of Rio de Janeiro, Rio de Janeiro-RJ, Brazil. mariliabgomes@uol.com.br** Disclosure Statement: **None

**Lois Jovanovic - **Sansum Diabetes Research Institute, Santa Barbara, California, U.S.A. ljovanovic@sansum.org **Disclosure Statement: **None

***For the Brazilian Diabetes Society (SBD) and Brazilian Federation of Gynecology and Obstetrics Societies (FEBRASGO)***.
